# Obstetric fistula policy in Nigeria: a critical discourse analysis

**DOI:** 10.1186/s12884-018-1907-x

**Published:** 2018-06-27

**Authors:** Oluwakemi C. Amodu, Bukola O. Salami, Magdalena S. Richter

**Affiliations:** Faculty of Nursing, Edmonton Clinic Health Academy, University of Alberta, Level 3, 11405 87 Avenue NW, Edmonton, AB T6G 1C9 Canada

**Keywords:** Obstetric fistula, Critical discourse analysis, Nigeria, Policy, Midwifery

## Abstract

**Background:**

In 2012, Nigeria’s Federal Ministry of Health published its National Strategic Framework for the Elimination of Obstetric Fistula (NSFEOF), 2011–2015. The framework has since lapsed and there is no tangible evidence that the goal of eliminating obstetric fistula was met. To further inform future policy directions on obstetric fistula in Nigeria, this paper explores how the NSFEOF conceptualized obstetric fistula and its related issues, including child marriage and early childbearing.

**Methods:**

A critical discourse analysis of the policy was performed. We examined four policies in addition to the strategic framework: the Nigerian constitution; the Marriage Act; the Matrimonial Causes Act; and the National Reproductive Health Policy. We used the three phases of critical discourse analysis: textual analysis, analysis of discourse practice, and analysis of discursive events as instances of sociocultural practice.

**Results:**

The analysis demonstrates that, despite its title, the policy document focuses on reduction rather than elimination of obstetric fistula. The overall orientation of the policy is downstream, with minimal focus on prevention. The policy language suggests victim blaming. Furthermore, the extent to which subnational stakeholders in government and civil society were engaged in decision-making process for developing this policy is ambiguous. Although the policy is ostensibly based on principles of social justice and equity, several rhetorical positions suggest that the Nigerian constitutional environment and justice systems make no real provisions to protect the reproductive rights of girls in accordance with the United Nations’ “2030 Agenda for Sustainable Development.”

**Conclusion:**

This analysis establishes that the Nigerian constitution, justice environment and the obstetric fistula policy itself do not demonstrate clear commitment to eradicating obstetric fistula. Specifically, a clear commitment to eradicating obstetric fistula would see the constitution and Marriage Act of Nigeria specify an age of consent that is consistent with the agenda to prevent obstetric fistula. Additionally, a policy to end obstetric fistulas in Nigeria must purposefully address the factors creating barrier to women’s access to quality maternal healthcare services. Future policies and programs to eliminate obstetric fistulas should include perspectives of nurses, midwives, researchers and, women’s interest groups.

## Background

The most severe long-term complication of prolonged, obstructed labour during childbirth is obstetric fistula. Obstetric fistula is an abnormal hole in the vaginal wall which links into the bladder (vesico-vaginal fistula) or the rectum (recto-vaginal fistula) or both [1]. This condition results when labour is prolonged and the presenting foetus becomes impacted within the birth canal. The vaginal soft tissue is compressed against the bony pelvis of the woman during labour and, if there is no timely intervention (such as emergency caesarian section), the vaginal tissue becomes necrotic. The necrotic tissue sloughs off, usually within three to ten days postpartum, and a hole develops in the birth canal which subsequently results in uncontrollable leakage of urine and/or feces through the vagina. This condition may be further complicated by infection, vaginal ulcers, scarring, and stillbirths, which are seen in 78 to 95% of cases [2–4].

Obstetric fistula disproportionately affects women and girls in low-resourced parts of the world. While precise figures are not available, it is estimated that more than 2 to 3.5 million girls and women live with untreated obstetric fistula with an added incidence of 50,000 to 100,000 new cases each year, mostly occurring in sub-Saharan Africa and Asia [5]. Obstetric fistula continues to disproportionately affect the most globally impoverished women and girls [5, 6]. Obstetric fistula has been a maternal health concern in Africa since 2050 BC when physicians of ancient Egypt first reported the condition in literature [7]. The continued existence of this condition, particularly in sub-Saharan Africa, represents the failure of the global community to address the sexual and reproductive health rights of women and girls, and to achieve equity in the distribution and access to reproductive health resources. Rates of obstetric fistula in Nigeria, are high, accounting for half of the worldwide cases [8]. Not coincidentally, Nigeria has the highest population of child brides in Africa (that is, girls married at 18 years of age and younger) [9]. Approximately 43% of girls marry before the age of 18 and 17% marry before the age of 15. Child marriage is more popular in the North West region of Nigeria, with figures as high as 76% [10].

## Context of maternal health policy in Africa

The current intervention schemes to eradicate obstetric fistula in Africa are historically linked to two landmark conferences. In 1987, at an international conference in Nairobi, the Safe Motherhood Initiative was launched to address the appalling state of maternal health in developing countries. The conference set a goal to reduce maternal mortality by three-quarters between 1990 and 2015. The second conference, the International Conference on Population and Development (ICPD), was held in Cairo, Egypt, in 1994. This conference declared that women’s and girls’ reproductive rights were at the heart of sustainable development. When the UN Millennium Development Goals (MDGs), particularly MDG 5 (improve maternal health), were adopted in 2000, many of them were premised on ICPD principles, prominent part of which included the elimination of obstetric fistula in Africa [11–13]. The eight MDGs were meant to be time-bound, achieved by 2015; however, MDG-5, improving maternal health, has not been accomplished in many African countries, and obstetric fistula persists [5, 8]. In 2003, the Fistula Care Project began conducting a six-year, facility-based needs assessment and policy intervention project in nine sub-Saharan African countries. Supported by the United Nations Population Fund (UNFPA) and implemented by EngenderHealth, in partnership with IntraHealth International, the assessment was intended to understand the causes and impact of obstetric fistula, country capacity to manage obstetric fistula and to identify clinical and programmatic gaps [1, 14]. The assessment demonstrated that although fistula prevalence can be reduced through surgical management, there are other complex determinants in the setting where women live such as poor referral and transportation system and poor access to perinatal support services that influence prevalence rates of obstetric fistula and preclude uptake of fistula treatment [14]. United Nations agencies and partners then encouraged each country to take ownership of their respective policy agenda for the eradication of obstetric fistula [15]. This meant integrating the policy on obstetric fistula within broader national development plans and poverty-reduction strategies of African nations. The ultimate goal of this agenda was to establish a country-specific “levels of care” model, where prevention is factored into all levels of the health system in each country [15, 16]. Nigeria was one of the first countries to implement such a model. Although policy was developed to eliminate obstetric fistula in Nigeria, obstetric fistula continued to be prevalent as of 2015 [5, 8]. Moreover, there was no comprehensive strategy to estimate progress made or to evaluate community ownership of the interventions within the sociopolitical context in Nigeria [14]. Likewise, country-specific obstetric fistula initiatives were modelled on the earlier Safe Motherhood Initiative and MDG-5 principles, which basically focus on technical improvement in biomedical maternal and obstetric care services and on awareness and education about reproductive health services [12].These initiatives were vertical, running parallel to local political and leadership structures within the countries; hence, there was no true ownership of the initiatives, and capacity building for sustainability was not achieved [12, 14].

In the UN General Assembly report on ending obstetric fistula published in 2016, it was identified that the three most cost-effective strategies to address obstetric fistula are the implementation of skilled birth attendance, emergency obstetric and newborn care and family planning [17]. Approaches developed in isolation of these manifold prerequisites are relatively ineffective [15, 17]. Also, midwives have a crucial role to reduce the high number of maternal disability from obstetric fistula [17]. While global strategy on addressing obstetric fistula continues to shift in theory, the administration of strategies is generically problematic at the national level because there is partial understanding of the structural, social and political conditions intersecting affected women’s experiences. These conditions include maternal healthcare access challenges, poverty, marginalization, gender and sociocultural inequality, barriers to education, especially for girls, child marriage and adolescent pregnancy [15, 17], 3. p. Prevention and treatment of obstetric fistula contributes to achieving Sustainable Development Goal 3, which is ensuring healthy lives; specifically, improving maternal health [17]. The agenda puts strong emphasis on country leadership and highlights the need to strengthen accountability through monitoring national progress and strengthening capacity to collect, analyze and use fistula program data. Nigeria’s obstetric fistula policy was one of the first strategic plans in West Africa and has been a model for other African countries. Taking Nigeria as a case study, this paper presents an analysis of how social and political systems are implicated in the problem of obstetric fistula.

### Nigeria

Nigeria is the most populous country in Africa with about 180 million people [18]. Although Nigeria constitutes only 2% of the world’s population, it accounts for 10% of the world’s maternal mortality rate in childbirth [19]. Maternal health initiatives began in Nigeria in 1990 after the Nairobi conference when Nigerian attendees organized a national Safe Motherhood conference convened by the Society for Obstetrics and Gynecology in Nigeria. After the country transitioned from military to civilian rule in 1999, a precarious political space was created to address some of the social issues related to maternal morbidities and mortalities. However, this transition concurrently diffused political power, making it easier to siphon public funds into private purses [20, 21]. Global attention was drawn to the problem of obstetric fistula in Nigeria in 2003 when UNFPA launched the landmark Campaign to End fistula. The initiative largely focused on improving treatment supplies and supporting existing fistula care centers. In 2005, the UNFPA began collaboration with the International Society of Obstetric Fistula Surgeons (ISOFS) to develop the National Strategic Framework for Eradication of Fistula (NSFEOF). The resulting guideline was an important initial step in the establishment of a cohesive obstetric fistula taskforce in Nigeria. ISOFS further grounded the strategy to address obstetric fistula by simultaneously formulating a competency-based training manual for fistula repair experts. The goal of the Strategic Framework was to reduce the incidence of obstetric fistulas by 80% and to have a 300% increase in fistula repair procedures between 2005 and 2010 [22]. Also during this period, a community screening module for detecting obstetric fistula symptoms was developed in Nigeria; and incorporated into the Nigeria Demographic and Health Survey to produce a community-based prevalence estimate. Within a decade, fistula treatment rate increased from 2000 repairs in 2003 to 6000 in 2013 [23]. In spite of progress in fistula treatment, there was evidence of continued inequity in access to maternal health care, reproductive rights, and distribution of social resources for women, which together constitute key factors in the aetiology of obstetric fistula [24].

After the initial framework lapsed in 2010, Nigeria’s federal government structured a new policy to eliminate obstetric fistula. The policy, titled *National Strategic Framework for the Elimination of Obstetric Fistula in Nigeria (2011–2015)*, was developed to increase access to prevention, treatment, and rehabilitation services. The priority focus of the policy was to deal with the underlying determinants of obstetric fistula [25]. Although the new policy initiative was ostensibly focused on elimination of obstetric fistulas in Nigeria, there is no tangible evidence to suggest that this goal was achieved [26]. The persistence of obstetric fistula and comorbidities in Nigeria [26] suggests that there is failure to truly integrate the lapsed obstetric fistula policy into national health and human rights initiatives, and to address the underlying determinants of this maternal health problem. The global agenda for obstetric fistula is to strengthen country-specific frameworks and reinforce national ownership, sustainability, and accountability for obstetric fistula policy development and problem solving toward 2030. The goal to strengthen country ownership of obstetric fistula eradication mandate engenders the need for a critique of policy discourses related to obstetric fistula to understand gaps in policy and program development. In line with this objective, we present an analysis of the 2011–2015 framework for obstetric fistula in Nigeria [25] and supporting social and reproductive health policies. Our aim in this paper is to understand how the legislative environment and the National Reproductive Health Policy framework, on which the obstetric fistula policy (2011–2015) is premised, embody the goal of ending obstetric fistula in Nigeria. Using critical discourse analysis, we examine the language and dominant paradigms in these policies to understand how the reality of obstetric fistula in Nigeria is conceptualized. We are specifically concerned with how the dominant paradigms in the policy texts construct the social reality of affected women.

## Methods

A critical discourse analysis of the *National Strategic Framework for the Elimination of Obstetric Fistula, 2011–2015* was conducted. Critical discourse analysis is an approach that examines how textual language constitutes aspects of social reality [27]. Critical discourse analysis is used to observe how language is used to justify inequality. Hence, this method can provide knowledge on how policy context constitutes obstetric fistula situation in Nigeria [28]. The *NSFEOF*, 2011–2015 was written by the Vesico-Vaginal Fistula (VVF) Technical Working Group, under the purview of the Federal Ministry of Health. The policy is premised on existing national health development frameworks, the *National Reproductive Health Policy*. The guiding principles of Nigeria’s obstetric fistula policy are social justice and equity, as declared in the 1999 constitution of the Federal Republic of Nigeria. The *NSFEOF* is divided into six main sections: (1) an introduction to obstetric fistula in the context of maternal mortality and morbidity in Nigeria; (2) a situational analysis of obstetric fistula in Nigeria; (3) a problem statement and priority action areas for 2011–2015; (4) the expected results; (5) coordination and feedback mechanisms for tracking implementation; and (6) a monitoring and evaluation plan [25].

The analysis was conducted by a three-membered team following an iterative process including purposive selection of relevant policies, textual interpretation and discursive analysis. The first author is a graduate student who completed training as a nurse and midwife in Nigeria. The second author is a Nigerian Canadian with expertise in critical methodologies and the third author is a South African Canadian with expertise in women’s health in a global context. We exercised reflexivity and considered our positionality throughout the study. In our initial analysis of the micro or linguistic level of the main policy on elimination of obstetric fistula, we examined the policy discursively by identifying the ideas in the policy document that made certain perspectives persuasive. We jointly identified sets of rhetorical tools on which authoritative claims in the text were constructed and developed themes based on emphasis or de-emphasis of ideas [29]. We then performed discourse analysis, which involves deconstructive analysis of how the text produces situational meaning in intertextual contexts. At this level, we focused on word choices, semantics, and grammar as rhetorical techniques of meaning making. The texts themselves expose silence and power struggles and illuminate how discursive hegemonies are produced. At the macro level (dialectic analysis), we did a social policy analysis by looking broadly into four other policies that shaped the environment in which the main policy was enacted. We examined trends across these four policies: the Nigerian constitution; the Marriage Act; the Matrimonial Causes Act; and the National Reproductive Health Policy. These social policies broadened the understanding of the issues surrounding obstetric fistula in Nigeria.

## Results and discussion

Our critical analysis of the *National Strategic Framework for the Elimination of Obstetric Fistula, 2011–2015* in Nigeria generated four main themes: (1) overall, there are contradictions in the obstetric fistula policy goals; (2) the policy takes a downstream approach to obstetric fistula management; (3) the policy uses a victim blaming discourse and focuses on individual behavioural change as a solution to high rates of obstetric fistula; and, (4) there is stakeholder ambiguity.

### Contradictions in policy goals

#### Constitutional misalignment with obstetric fistula policy goals

Although the policymakers claimed that they consulted a plethora of policies in developing the *NSFEOF*, it is not clear how these other policies align with the goal of eliminating obstetric fistula. For example, although the Nigerian constitution supposedly provides a foundation of social justice and equity to support the *NSFEOF,* activists and political observers alike have questioned the constitution’s commitment to social justice. While the constitution states the “right to dignity of human persons” and the “right to personal liberty” in its chapter on “Fundamental Rights,” [30], the Nigerian constitution makes no reference to the prevention of child marriage—a cultural norm that has long violated these rights [10, 30]. Our findings demonstrate that there is overt silencing of the issue of forced marriage and early childbearing which is a strong determinant of obstetric fistula in Nigeria. Although women of all ages are affected by obstetric fistula, underdeveloped pelvises which is the case when girls are young or malnourished, is a major biological predisposing factor to obstetric fistula [16, 31]. In other words, the federal government is constitutionally detached from the responsibility of protecting the rights of minors, which complicates its role in the elimination of obstetric fistula, despite the downstream approach of the *NSFEOF* that gives primary authority to the federal government.

Another paradox arises in the constitutional efforts to protect girls from early marriage. In Nigeria’s constitution, the Sharia judiciary system presides over marriages at subnational levels, particularly in northern states of Nigeria. This means that the Sharia Court of Appeal is responsible for deciding “any question of Islamic personal law regarding marriage,” including the validity or dissolution of a marriage and questions about family relationships and the guardianship of infants [32]. This means that the constitution hands over control of women’s reproductive rights to the Sharia Court of Appeal in northern regions of Nigeria. Sharia law itself contradicts the constitution, specifically section 29(4) (b) of the constitution which states that “any woman who is married shall be deemed to be of full age” [33]. Sharia law permits the marriage of minors under certain circumstances which trumps this constitutional assertion that a girl who is married should be considered automatically of full age. Moreover, constitutional laws do not control regional regulations. In particular, northern states’ rulings on child marriage do not align with the federal constitution. In northern Nigeria, Islamist jurists believe that a minor can be married under Sharia law if she has attained menarche [30]. There is a concept known as *ij bar*, or “fatherly power,” where a father can force a daughter to be married [34, 35] (p. 10). Sharia law also states that men can take up to four wives while women can have only one husband [36]. Marriage can occur at any time but, according to Sharia law, consummation should only occur when the girl reached menarche [37]. Hence, girls are given in marriage to men with the hope that these men will wait until the girls are “of age” before they have sex, nevertheless some men have reportedly raped these child brides [30].

Although the Nigerian government passed the Child Rights Act, which sets the legal age for marriage at 18, in 2003, federal legislation is only effective if it is passed at the state level [30]. As of May 2016, only 23 of Nigeria’s 36 states have passed the act [38]. In 2016, a bill supporting gender parity and further legislative efforts to end child marriage met stiff resistance from the National Assembly of Nigeria on the grounds that gender equality contradicts Islamic traditions and culture [39]. The *NSFEOF* also employs the rhetoric of *“reproductive rights”,* which helps make the framework seem attentive to justice. However, the precise regulations that are being applied to accomplish justice within the *NSFEOF* is not clear. The framework refers specifically to the Nigerian Labour Law, the Marriage Act, the Matrimonial Causes Act, and the constitution, but it characterizes particular parts of these pieces of legislation as controversial: “Section 54 of the Nigerian Labour Law, Chapter 21 and Part 5 of the Criminal Code, and sections 18 of the Marriage Act as well as section 3 of the Matrimonial Causes Act contain relevant but controversial provisions related to reproductive health and rights” (p. 18). Although the *NSFEOF* does not explain what the controversies are, the use of the word *“controversial”* here alludes to Islamic objection to gender equality. The desire to avoid the *“controversial”* aspects of these laws means that the federal government can sidestep responsibility for legislating women’s reproductive rights.

The Marriage Act and the Matrimonial Causes Act reinforce a similar mismatch. For instance, neither the Marriage Act [40] nor the Matrimonial Causes Act [41] prescribes a specific minimum age of consent for the marriage of minors. They do give an age of consent (21) so that they appear to regulate the age of consent. However, under section 18 of the Marriage Act [41], there is no limit to the age of consent as long as there is parental approval. The rhetorical strategy is to offer a regulation that offers parental liberty to betroth daughters. The ambiguities concerning age of consent and minimum age for marriage in the Nigerian constitution and the Marriage Act provide evidence to suggest that that Nigerian legislative environment is inconsistent with an agenda to eliminate obstetric fistula. At the textual level the *NSFEOF* performs a disturbing discursive feat by rhetorically separating early childbearing from early marriage:

*“Possible factors in the formation of obstetric fistula include static gender norms…poverty, ignorance, illiteracy, preference for home delivery and the desire to avoid Caesarean section, early childbearing (as opposed to early marriage); harmful traditional practices like “gishiri cut,” (female genital cutting), low social status of women coupled with poor access to and utilization of EMOC services are other reasons proffered for the higher incidence of obstetric fistula in Nigeria.”* [25] (p. 22).

### Downstream approach to obstetric fistula management

Like many downstream health interventions, which involve individual-level behavioural approaches for prevention or management of illness, the Nigerian obstetric fistula framework focuses on the treatment of existing cases, despite its claim to be concerned with prevention and rehabilitation as well. The framework measures success in terms of the *“number of repairs”* [25] (p. 25), whereas an upstream approach might measure success in reducing rates of poverty, for example, as one of the social determinants of health that affects rates of obstetric fistula. In the foreword, the minister of health makes the policy’s downstream approach clear: *“the goal of this document is to provide a standard reference material that can be used to train health workers and also guide them in the provision of holistic, respectful, simple, affordable, quality and evidence-based care for obstetric fistula women that will guarantee improved quality of life for these women and their families”* [25] (p. 2). The phrase *“obstetric fistula women”* emphasizes that this framework was designed not to address the social realities of women who are at risk for developing obstetric fistula, but to treat obstetric fistula in the women who are already experiencing it. On the other hand, the literature establishes that maternal health services are weak in Nigeria. Antenatal care coverage in Nigeria is around 66% and, institutional child birth and contraceptive prevalence rate falls well below 50% [17]. This shortage in maternal health services heighten women’s risk to obstetric fistula in Nigeria. While improved medical management of fistula does enhance the lives of affected women [24, 42], there are ambiguities in the long-term appropriateness of discrete curative approaches to the broader mission of eliminating obstetric fistula in Nigeria. For example, in Nigeria, fistula repair camps are conducted intermittently where certain numbers of fistulas are repaired [42]. Although these fistula treatment sites are a promising strategy for reducing the number of women awaiting fistula treatment, this method does not suffice for preventing new cases of fistula. Moreover, evidence suggests that many of the trained repair experts tend not to be committed to this field of fistula treatment for reasons related to professional development. In addition, these short-term medical treatment campaigns often do not reach women in more isolated, rural communities, which reflects the inequality in access for rural women [43]. In many cases, the women’s voices are never heard because of the shameful and marginal circumstances they find themselves [44]. There is no data to suggest that the elimination of obstetric fistula was the *NSFEOF*’s actual goal. In fact, the discrepancy between the title’s professed goal of the *“elimination of obstetric fistula”* and the content’s explicit aim for a *“reduction”* of obstetric fistula incidence by 50% from current level [25] (p.9), suggests that the strategy to eliminate obstetric fistula was grandiloquent and unrealistic.

### Victim blaming discourse and behaviour change strategies

In the *NSFEOF*’s Executive Summary, access to appropriate care is emphasized as a key issue in addressing obstetric fistula: *“Improving service delivery by increasing the uptake of family planning, delaying marriage and early births, increasing access to quality maternal health services are also important in the national response”* [25] (p. 8). It is difficult to comprehend how increasing *“uptake”* translates to improving *“service”* delivery. This statement shifts the focus from the service delivery as an issue, to service uptake by women, making it appear that the unavailability of health service is somehow a result of women not accessing the services. According to this statement, demand of maternal services is a problem of supply. This is a form of gerrymandering gone wrong, fallible on many fronts. It is well known that Nigerian women’s needs grossly outweigh the available services [22, 45]. In addition, many women do not patronize medical services because of the unsatisfactory antenatal and obstetric care they receive from grossly under-motivated staff particularly working in rural areas [45, 46].

Furthermore, the policy equates low levels of formal education with women’s ability to access maternity services. The language used in the executive summary: *“obstetric fistula woman”* suggests stereotyping, as well as a focus on women who already have obstetric fistula rather than targeting those at risk also. The use of terms such as “obstetric fistula woman,” “uneducated,” and “ignorance” serves to pathologize women and undermine their knowledge and voice, suggesting that inherent intellectual deficit is at the core of their obstetric fistula calamity. This language connotes subjection, blames the victim, and negates other factors in women’s lives that contribute to obstetric fistula. Victim blaming can obscure the unique experiences of affected women, and can be an effective tool used by government to focus intervention on the affected woman as the one who needs a behavioural change, while entrenched administrative shortcomings are deemphasized. By labeling an already oppressed group with more stigmatizing terms, attention shifts from broader facets of the problem to a behavioural change focus of intervention [25]. Newer studies challenge this common instrumental narrative of women with obstetric fistula [47–49]; they suggest a need to look into the problem of obstetric fistula purposefully to describe it contextually, rather than to label it. That is, to explore the economic, political, moral and religious dimensions of the problem. Additionally, in the *NFSEOF* and the reproductive health policy in Nigeria, behavioural change communication is referenced as a focus of policy initiatives [25] (p. 34), yet, there is failure to identify an explicit strategy for capacity building of frontline staff, particularly nurses and midwives, for implementing behaviour change programs for women at grassroots levels in Nigeria. Discrete behavioural change interventions have become a gospel of “safe motherhood” for Africa [50–52] and is long-established as a toothless strategy when it comes to dealing with the systemic issues—namely, the forces of paternalism in government, in law and in health systems that downplay the issue of maternal health access and sustain reproductive rights abuse of girls and women. Nigeria may be wasting public funds on behaviour change interventions for women on a path to eliminate fistula while structural limitations such as lack of skilled birth attendance and practices of child marriage remains so prevalent and are maintained by cultural and political hegemonies [53].

### Stakeholder ambiguity

The seventh of ten overarching *NSFEOF* principles reads, *“Since health is an integral part of overall development, inter-sectoral cooperation and collaboration between the different health-related Ministries, development agencies and other relevant institutions shall be strengthened”* [25] (p. 31). Despite a commitment to inter-sectoral collaboration, the VVF Technical Working Group relied disproportionately on the Federal Ministry of Health and the philosophy of its national health plan, which espouses health paradigms that ignore the impact of broader social policies on health, particularly in the case of obstetric fistula. The *NSFEOF* describes the national health plan as *“developed in a fully participatory manner that involved all the key stakeholders in health—Federal, State, Local government, international and domestic partners, and civil society organizations”* [25] (p. 19). In this description, civil society is notably subsumed under the umbrella of “stakeholders in health”; thus, references to *“civil society”,* which occur throughout the policy, implicitly refer back to the vaguely defined “stakeholders in health.” Moreover, the term *“fully participatory”* is bombastic rhetoric that appears inclusive of grassroots sectors but in practice defines stakeholders as those within the professionalized sectors of health and global development. Therefore, the question arises, how do Nigerian sub-national governments and communities of citizens influence the health sector and development agencies’ decision making?

The lack of specificity of the role of civil society organizations is further shown by the *NSFEOF*’s emphasis on the centrality of the Federal Ministry of Health: *“In the last 5 years of implementation of this lapsed strategy [the previous five-year plan], the Federal Ministry of Health remained in the lead in coordinating the work of multiple actors at the Federal, State, Local levels, Civil Society and international partners”* [25] (p. 13). With this statement, the *NSFEOF* makes it clear that the Ministry of Health is the primary authority; it holds the power to regulate the administration of the framework. Hence, civic participation may not imply public sector participation as control of policy operations is maintained by the federal government and the Federal Ministry of Health. Although the obstetric fistula policy claims to embrace a social justice model, the tone of the policy is discursively palliative rather than emancipatory.

## Implications

Taking into account the incoherence of the policy goals and the fact that there is no clear alignment of obstetric fistula policy goals with the constitutional and customary law on reproductive rights of girls, the legislative environment on which the *NSFEOF* was founded is contentious. Obstetric fistula is more than a health problem. It is a social problem related to women’s oppression and the rights abuses of girls and women. Addressing obstetric fistula necessitates, first, a systematic deliberation on how the vulnerability and subjectivity of women and girls are constructed in the intermeshing political and cultural practices in Nigeria that predate the new global agenda of reproductive rights and gender equality. Within Nigeria, future obstetric fistula policies need to implicate broader social policy and constitutional laws. The policy to end obstetric fistula needs to be aligned with other national health policies and the constitution of Nigeria. Practical steps to implement the Child Rights Act should be taken to protect the rights of girls and women. According to former UN Secretary-General Ban Ki-moon, girl child marriage and gender-based violence are obstructing SDG-5—to achieve gender equality and empower all women and girls by 2030 [54]. Female education should become priority in Nigeria and there needs to be equal gender representation in leadership positions and in judiciary. Given the emphasis on medical remedies and fistula repair coupled with the sidelining of the role of nurses and midwives, we can conclude that the biomedical paradigm dominates the *NSFEOF*. Biomedical perspectives are essential for the provision of repair services for women affected by obstetric fistula in Nigeria. However, policy makers must concurrently recognize that access to quality maternal health services for women in Nigeria are equally critical to the prevention and elimination of obstetric fistula. The issue of shortage of quality maternal health services should have equal consideration when policy is formulated on obstetric fistula in Nigeria.

In the *NSFEOF*, there is only minimal acknowledgment of women as stakeholders, able to resist social injustices and oppression. Affected women are stereotypically portrayed as passive victims, sexually bound to men, and thoroughly oppressed. However, research needs to look beyond these stereotypes to understand the resilience and power of women for creating new definitions of female emancipation. A review of social policies in Africa needs to be done to better situate an obstetric fistula strategy within a national development agenda and to mobilise political and health systems to align activities with the goal of eliminating obstetric fistula. The fistula taskforce will benefit from the inclusion of nurses and midwives who bring research experience based on critical feminist methodologies to shed light on women’s experiences as shaped by broader reproductive rights issues. In particular, perspectives that focus on addressing structural determinants of health inequities for women is necessary. These determinants include public policy shaping the maternity care subsidy available to women during childbirth and the need to upgrade standards of maternity services at primary health centers [22]. Accessibility to maternity services and fistula care needs to account for the disparity in the wealth index of the country. Obstetric care services should be concentrated in areas where women are least likely to be able to reach care. The focus of funding commitments ought to shift to preventing obstetric fistula by developing capacity within the primary health care institutions. Primarily, there is need for expansion of community-based nursing and midwifery services for women in rural areas to ensure safety during childbirth for all women in Nigeria.

## Conclusion

UNFPA recommends individual country ownership of policies to end obstetric fistula in Africa. Nigeria was one of the first African countries to develop a policy to end obstetric fistula. The UN initiative to have country ownership of obstetric fistula policies has kicked off without clear alignment between the UN initiative and individual countries’ policies. In the case of Nigeria, it is clear that the health systems and the legislative environment are not prepared to accommodate a comprehensive agenda to end obstetric fistula. Analysis of the *NSFEOF*, suggests an overarching dichotomy between the national obstetric fistula framework and the local policy context in Nigeria. In particular, there is minimal acknowledgment of systemic problems including defective social policies and relations of political power that multiply the obstetric fistula menace in Nigeria. Moreover, the *NSFEOF* took a downstream approach to obstetric fistula, with authority concentrated in the federal government and affiliated institutions. There was ambiguity in the level of participation of civic stakeholders and contradictions in policy goals.

Contradictions between constitutional law and customary law especially on the protection of the reproductive health and rights of girls on the path to preventing early childbearing were evident. For instance, the language of the *NSFEOF* isolates early marriage from early childbirth in the Nigerian context intentionally ignoring the relationship between forced marriages and early childbearing. The Nigerian constitution and the Marriage Act thus, systematically promote early child marriage and by extension, early child bearing. Early child marriage is a precursor to early childbearing, and these events should not be portrayed as separate variables within the discussion of the causation of obstetric fistula among young girls, especially within a framework of social and reproductive justice. For interventions to end obstetric fistula to be effective and sustainable, priority prevention strategy should be the enforcement of laws against child marriage and for development of policy to educate and empower girls and women in Nigeria. While the *NSFEOF* framework aims to address obstetric fistula as a rights issue, strategies are limited to behaviour change model of intervention with further emphasis on biomedical solutions and less emphasis on preventative and community-grounded strategies. To inform a more holistic approach, future obstetric fistula policy development taskforce should be decentralized and engage midwives, nurses, researchers and, women’s interest groups as stakeholders with a voice for advocacy. Drafting lofty policies to eliminate obstetric fistula without focusing on implementing quality maternity services for women during childbirth is counter intuitive. At all levels, quality maternal health services need to be provided for all women to support safe childbirth in Nigeria.

## Abbreviations

ISOFS: International Society of Obstetric Fistula Surgeons
MDG: Millennium Development Goals
NSFEOF: National Strategic Framework for the Elimination of Obstetric Fistula
SDG: Sustainable Development Goal
